# Associations of Atrioventricular Blocks and Other Arrhythmias in Patients with Lyme Carditis: A Systematic Review and Meta-Analysis

**DOI:** 10.3390/jcdd11050131

**Published:** 2024-04-23

**Authors:** Nismat Javed, Eduard Sklyar, Jonathan N. Bella

**Affiliations:** 1 BronxCare Health System, Bronx, NY 10457, USA; nismatjaved@gmail.com (N.J.); esklyar@bronxcare.org (E.S.); 2 Icahn School of Medicine at Mount Sinai, New York, NY 10029, USA

**Keywords:** Lyme carditis, atrioventricular blocks, outcomes, management

## Abstract

Lyme disease often leads to cardiac injury and electrophysiological abnormalities. This study aimed to explore links between atrioventricular blocks and additional arrhythmias in Lyme carditis patients. This systematic review and meta-analysis of existing literature was performed from 1990 to 2023, and aimed to identify cases of Lyme carditis through serology or clinical diagnosis with concomitant arrhythmias. Pubmed and Web of Science were searched using appropriate MESH terms. Patients were divided into groups with atrioventricular blocks and other arrhythmias for cardiovascular (CV) outcome assessment. A total of 110 cases were analyzed. The majority (77.3%) were male, with mean age = 39.65 ± 14.80 years. Most patients presented within one week of symptom onset (30.9%). Men were more likely to have first-degree atrioventricular blocks (OR = 1.36 [95% CI 1.12–3.96], *p* = 0.01); these blocks tended to be reversible in nature (OR = 1.51 [95% CI 1.39–3.92], *p* = 0.01). Men exhibited a higher likelihood of experiencing variable arrhythmias (OR = 1.31 [95% CI 1.08–2.16], *p* < 0.001). Ventricular and supraventricular arrhythmias were more likely to exhibit instability (OR = 0.96 [95% CI 0.81–1.16] *p* = 0.01) and variability (OR = 1.99 [95% CI 0.47–8.31], *p* < 0.001). Men with Lyme carditis are likely to present with various atrioventricular blocks. These atrioventricular blocks are benign, and follow a predictable and stable clinical course. Further large-scale studies are warranted to confirm these associations.

## 1. Introduction

Lyme disease is a prevalent global vector-borne illness, particularly in Europe, Eurasia, and the US. In Europe and Eurasia, the yearly incidence rate is approximately 0.21%, with higher rates in specific areas like northeast Moscow and Western Siberia [1,2,3,4]. In the US, between 2008 and 2015, the CDC documented 208,834 confirmed cases and 66,755 probable cases, with July being the peak month for onset [5]. Lyme disease can lead to cardiac complications, with about 1% of patients developing Lyme carditis, typically between June and December of the year following exposure [6,7]. This condition can manifest as atrioventricular block or left ventricular dysfunction, along with ECG abnormalities like T wave irregularities and prolonged QT intervals [8,9,10,11,12]. While antibiotics are effective in treating Lyme disease, they seem less critical in reversing cardiac manifestations, suggesting inflammation as a driving factor in Lyme carditis [13,14,15]. However, data on risk factors and treatment for Lyme carditis remain limited, highlighting the need for further research.

The objective of the study was to determine associations of atrioventricular blocks and other arrhythmias in patients with Lyme carditis. The study also focuses on demographic characteristics, clinical presentations, treatment, and outcomes of both cohorts with Lyme carditis.

## 2. Materials and Methods

### 2.1. Protocol Development and Search Strategy

The protocol was registered in PROSPERO (ID# CRD42023448732) and followed the guidelines of the Preferred Reporting Items for Systematic Reviews and Meta-Analyses (PRISMA) [16]. We implemented the patient, intervention, comparison, and outcomes (PICO) approach for our research query in this study. The keywords employed were: “lyme” [All Fields] AND (“myocarditis” [MeSH Terms] OR “myocarditis” [All Fields] OR “carditis” [All Fields]) AND (“arrhythmia s” [All Fields] OR “arrhythmias, cardiac” [MeSH Terms]) OR (“arrhythmias” [All Fields] AND “cardiac” [All Fields]) OR (“cardiac arrhythmias” [All Fields] OR “arrhythmia” [All Fields] OR “arrhythmias” [All Fields]).

### 2.2. Data Extraction

**Figure 1:**
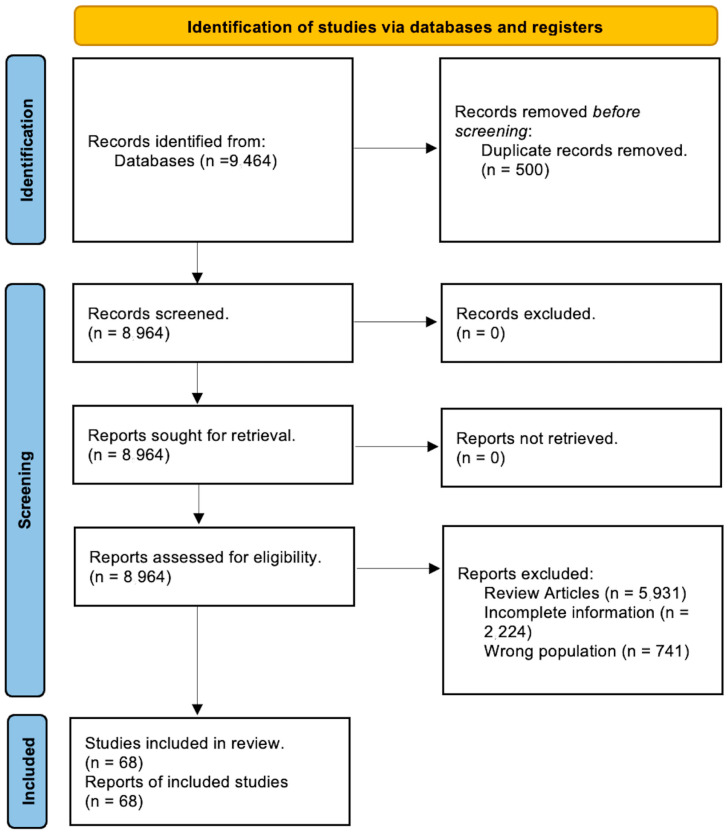
PRISMA diagram reviewing all studies.

Our search was conducted on PubMed and Web of Science using Boolean operators (“OR”, “AND”). This systematic review incorporated articles written in English over the past three decades that met the following inclusion standards: (1) articles that addressed Lyme carditis or arrhythmias in patients with a confirmed Lyme disease diagnosis via serology or clinical symptoms; and (2) articles that specifically detailed interventions used for patients with arrhythmias in Lyme carditis and their results. Employing the PICO approach, we evaluated patients diagnosed with Lyme carditis. The interventions referred to the treatment strategies applied (such as antibiotic treatments and pacemaker implantation). The outcome variables included mortality, arrhythmia reversibility, clinical symptoms, and reversibility duration, which were compared with patients without AV block arrhythmias. To reduce population bias and enhance qualitative variable measurements, the systematic review included observational studies and case reports. The initial search yielded 164 articles from PubMed and 9300 articles from Google Scholar. After removing duplicates, two independent reviewers screened the remaining studies based on the inclusion criteria, initially reviewing abstracts and then full-text articles if criteria were met (N.J. and J.N.B.). Zotero (version 6) and Rayyan (version 2016) software were used for this process. A third reviewer was brought in to resolve any possible disputes (E.S.). One reviewer (N.J.) extracted details about the study design, publication year, study location, journal of publication, demographic data, initial symptoms, diagnostic characteristics, types of arrhythmias, management, and outcomes from the records. The collected data was then double-checked for its accuracy and completeness. Studies were excluded from the analysis if translations or complete articles were unavailable, or if the information about device-specific characteristics was incomplete. Non-peer-reviewed papers were also omitted. The initial data was logged into an Excel spreadsheet, and the final analysis included 68 studies. These findings are illustrated in Figure 1.

STATA version 18 was used to perform the statistical analysis. Continuous variables were expressed as mean ± standard deviation. Qualitative variables were represented by frequencies or percentages. Statistical tests such as chi-square, independent t-test, and analysis of variance were employed. To identify predictors for multiple outcomes, logistic regression models were utilized. A *p*-value of less than 0.05 was deemed significant.

## 3. Results

### 3.1. Demographic and Clinical Characteristics

The analysis encompassed a total of 68, comprising 5 case series and 63 case reports. The cumulative number of cases considered in the review amounted to 110. Predominantly, the cases were male, accounting for 78.10% of the total. The mean age of the study population was 39.65 ± 14.80 years. Detailed information regarding data extraction from the articles is provided in Table S1 [17,18,19,20,21,22,23,24,25,26,27,28,29,30,31,32,33,34,35,36,37,38,39,40,41,42,43,44,45,46,47,48,49,50,51,52,53,54,55,56,57,58,59,60,61,62,63,64,65,66,67,68,69,70,71,72,73,74,75,76,77,78,79,80,81,82,83,84]. Most commonly, patients had presented with electrophysiological abnormalities within 2 weeks of initial symptoms (40.9%). Prior comorbidities had been discussed for 27 cases; hypertension was present in 13.6% of the cases. Syncope was present in 32/110 cases (29.1%), followed by palpitations in 14/110 cases (12.7%) and dizziness in 8/110 cases (7.3%).

### 3.2. Laboratory Investigations, Electrocardiogram- and Echocardiogram-Based Features

As can be seen in Table 1, most of the cases were hemodynamically stable on presentation (104/110 cases). Echocardiographic findings were not documented for many cases (55/110 cases).

Troponinemia was observed in 4/110 cases. Increased inflammation was observed in 9/110 cases. Diastolic dysfunction was observed in 11/110 cases. Systolic dysfunction was observed in 5/110 cases. Most of the patients presented with first-degree (25/110) or complete atrioventricular block (24/110).

### 3.3. Management

As may be seen in Table S2, most of the cases were managed with antibiotics (70/110; 63.6%). Pacing was needed for 22/110 cases. The arrhythmias resolved with treatment in 88/110 cases. Six patients died during the course of illness.

### 3.4. Subgroup Analysis

The subgroup analyses are detailed in Table 2 and Table 3.

Men were more likely to present with syncope, had a shorter time to resolution, and presented with variable arrhythmias. However, atrioventricular blocks were likely to be stable and reversible. Patients with atrioventricular blocks were likely to present with syncope and had a shorter time to resolution. The abnormalities were more stable and reversible in nature. However, the type of atrioventricular blocks varied.

## 4. Discussion

Our study had a few key findings. First, the majority were young men with mean age = 39.65 ± 14.80 years. Male sex has been identified has one of the independent predictors of developing Lyme carditis [85,86,87]. In the US, men and women have been found to have similar odds of developing Lyme disease (50% in men and 50% in women) [88]. In European studies, a preponderance for the female population in developing Lyme disease has been noted. As per the literature, these differences could not be explained by attire, usage of deodorant/perfume, or activities at the time of the tick bite [89]. One of the hypotheses suggested in the study included a variation in neurochemical hormones that leads to varying rates of tick bites [89]. However, this has not been discussed in the literature. Even in studies with relatively equal gender distributions, the risk of developing carditis was higher in men [90]. In our study, younger individuals were at a higher risk of developing Lyme carditis. However, it was not an independent predictor of development of cardiac abnormalities. This is similar to another study mentioning older age as one of the independent predictors for Lyme carditis [86], particularly due to an increase in comorbidities.

Second, the majority of the patients had presented with atrioventricular blocks (50.9%). A few hypotheses can be considered for this finding, as described by previous models. One of the borrelial proteins, P66, binds to the integrin receptors, resulting in greater extracutaneous dissemination of bacteria and cardiac tropism [91]. Similarly, one of the proteoglycans called decorin-binding protein causes colonization of the heart. However, this impact has been studied in murine models, and more investigations are needed to determine the long-lasting effects of the proteins [92,93]. Men were more likely to have atrioventricular blocks that were stable and reversible. As observed in another study, men had a longer AV block cycle in supraventricular arrhythmias (371 ± 76 ms vs. 330 ± 52 ms, *p* = 0.02) compared to women [92]. More ventriculoatrial dissociation was observed in men [94]. Additionally, the previous literature also suggests that men have a preponderance to develop atrioventricular block regardless of age, as discussed in a Chinese study. Men were 2.44-fold more likely to develop atrioventricular block after adjusting for age [95]. This implies that, in men, PR interval representing both autonomic and structural cardiac abnormalities indicated progressive fibrosis, which is also a measure of advanced age, might be hypothesized to be the causative factor [96].

Limited data exist on antibiotic guidelines for Lyme carditis management. UK guidelines recommend oral doxycycline or amoxicillin for stable patients, and intravenous ceftriaxone for those with hemodynamic compromise or systemic illness, typically for 14 to 21 days. Once-daily administration of ceftriaxone is advantageous [97], but most patients in our study were hospitalized regardless of atrioventricular block type, suggesting a need for revised guidelines. Apart from use of antibiotics, one algorithm discussed use of pacemaker implantation and subsequent explantation. Pacemaker implantation is common for symptomatic bradycardia, especially with high-grade AV blocks, as these are associated with cardiac arrest [7,98]. In Lyme carditis, brief periods of asystole are associated with a worse prognosis, along with escape rhythms featuring a wide QRS complex and fluctuating bundle branch blocks [99]. Pacemaker interrogation should be performed after completion of antibiotic therapy. In cases of refractory ventricular pacing, the pacemaker is usually long-term. In other cases, the Wenckebach point comes into play, with heart rates <80 beats per minute warranting long-term pacemaker placement [100].

Three patients with atrial fibrillation and first-degree atrioventricular block died, while those remaining had associated ventricular arrhythmias; limited data explain this distribution, but prior studies suggest that men with ventricular arrhythmias have a worse prognosis [100].

The main strengths of the study include a gender-based comparison of disease pattern in Lyme carditis, as well as a comparison of the electrophysiological abnormalities. There are a few limitations to the study. Men were in higher numbers in the study, which can produce bias in analysis, despite being adjusted for. Therefore, these findings need to be interpreted in appropriate context. Multiple studies used qualitative measures that could not be compared in a pair-wise fashion, despite producing significant results in the studies discussed. Data about prior histories of comorbidities and echocardiographic findings were limited.

## 5. Conclusions

The study explored the relationship between Lyme disease and cardiac complications, specifically focusing on atrioventricular blocks and other arrhythmias in Lyme carditis patients. Men were more likely to develop first-degree atrioventricular blocks, often reversible in nature. Additionally, men showed a higher likelihood of experiencing various arrhythmias, with ventricular and supraventricular arrhythmias associated with instability and variability. The study suggests a potential predisposition of men with Lyme carditis to atrioventricular blocks, highlighting the need for further comprehensive research to confirm these associations.

## Figures and Tables

**Table 1:** Features on presentation.

Authors	Vitals	Labs	Electrocardiogram	Echocardiogram
Wan et al. [17]	Stable	NA	3-complete block, 2-2:1 AV block	mild right ventricular dilation focal myocarditis and diastolic dysfunction. No LVEF reported
Zainal et al. [18]	Stable	Normal	Atrial fibrillation with RVR	No LVEF reported
Gazendam et al. [19]	Stable	Normal	Sick Sinus Syndrome	LVEF: 60%
Esfandiari et al. [20]	Unstable	High troponin	Ventricular Tachycardia > cardiac arrest	moderately reduced left ventricular systolic function (LVEF: 40–45%) with mild diffuse hypokinesis, abnormal septal motion, and a mildly dilated right ventricular cavity with mildly reduced systolic function.
Franco-Avecilla et al. [21]	Stable	Normal	1st degree AV block	No LVEF reported
Bamgboje et al. [22]	Stable	Normal	3rd degree AV block	LVEF: 60%
Zaid et al. [23]	Stable	Normal	Complete heart block	LVEF > 50%
Arroja et al. [24]	Stable	Leukocytosis	1st degree block	No LVEF reported
Kannangara et al. [25]	Stable	Normal	3rd degree AV block	LVEF: 60%
Rojas-Marte et al. [26]	Stable	Normal	AV disassociation and ventricular escape rhythm	No LVEF reported
Khetpal et al. [27]	Stable	Normal	Atrial flutter with slow ventricular response	No LVEF reported
Wang et al. [28]	Stable	Normal	Junctional rhythm	No LVEF reported
Yoon et al. [29]	Unstable	Leukocytosis, AKI	Ventricular Tachycardia	No LVEF reported
Dobbs et al. [30]	Unstable	Leukocytosis	Junctional rhythm	No LVEF reported
Beach et al. [31]	Stable	None	Paroxysmal junctional tachycardia	No LVEF reported
Shabbir et al. [32]	Stable	None	Atrial fibrillation	No LVEF reported
Kennel et al. [33]	Stable	None	Atrial fibrillation	No LVEF reported
Brunner et al. [34]	Stable	High troponin	Type II AV block	LVEF was normal
Greenberg et al. [35]	Stable	None	Fascicular tachycardia	No LVEF reported
Siebenlist et al. [36]	Stable	None	Complete heart block	No LVEF reported
Rostoff et al. [37]	Stable	None	Complete heart block	LVEF: 45%
Aringer et al. [38]	Stable	None	AV type 2 heart block	No LVEF reported
Aringer et al. [38]	Stable	None	AV type 2 heart block	No LVEF reported
Vasiljević et al. [39]	Stable	None	AV block type 2 (x2), AV block type 3	No LVEF reported Enlarged atrium
Panic et al. [40]	Stable	None	AV block type 2	No LVEF reported, Mitral and tricuspid regurgitation
Dam et al. [41]	Stable	None	AV block type 3	No LVEF reported
Manek et al. [42]	Stable	None	Complete heart block	No LVEF reported
Nutt et al. [43]	Stable	None	1st degree AV block	No LVEF reported
Khalil et al. [44]	Stable	None	Fascicular tachycardia	No LVEF reported
Mayer et al. [45]	Stable	None	Junctional rhythm	No LVEF reported
Jensen et al. [46]	Stable	None	Torsades de pointes	No LVEF reported
Jiménez-Castillo RA et al. [47]	Stable	None	3rd degree AV block	No LVEF reported
Lórincz et al. [48]	Stable	Leukocytosis	3rd degree AV block	No LVEF reported
Vlay et al. [49]	Stable	Leukocytosis	Ventricular Tachycardia	No LVEF reported, LVEDP high
Munk et al. [50]	Stable	Leukocytosis	3rd degree AV block	No LVEF reported, LVEDP high
Dernedde et al. [51]	Stable	High troponin, transaminitis	Supraventricular tachycardia	LVEF—38%
Isath et al. [52]	Stable	None	AV block	Complete regression of wall motion
Timmer et al. [53]	Stable	None	3rd degree AV block	No LVEF reported
Clinckaert et al. [54]	Stable	Elevated CRP and leukocytosis	3rd degree AV block	LVEF was Normal
Zande et al. [55]	Stable	Elevated CRP	2nd degree AV block	LVEF was normal
Matthiae et al. [56]	Stable	None	2nd degree AV block	No LVEF reported
Xanthos et al. [57]	Unstable	Elevated CRP and leukocytosis	1st degree AV block > 2:1 AV block > 3rd degree AV block	No LVEF reported
Rosenfeld et al. [58]	Stable	Elevated ESR	3rd degree AV block	No LVEF reported
Wenger et al. [59]	Stable	Elevated CRP	3rd degree AV block > asystole	No LVEF reported, mild to moderate mitral regurgitation
Bhattacharya et al. [60]	Stable	Elevated CRP and high troponin	Atrial fibrillation > AV block	No LVEF reported
Lo et al. [61]	Unstable	None	1st degree AV block	No LVEF reported
Semmler et al. [62]	Stable	None	3rd degree AV block	No LVEF reported
Chauhan et al. [63]	Unstable	None	3rd degree AV block	No LVEF reported
Franck et al. [64]	Unstable	None	1st degree AV block	LVEF: Normal
Brownstein et al. [65]	Stable	None	Sinus arrest	LVEF: Normal
Prochnau et al. [66]	Stable	Leukocytosis	1st degree AV block with ventricular asystole	LVEF: Normal
Semproni et al. [67]	Unstable	Elevated D-dimer and CRP high	1st degree AV block	LVEF: Normal
Konopka et al. [68]	Stable	Elevated lactate and metabolic acidosis	3rd degree AV block > PEA	No LVEF reported
Marx et al. [69]	Unstable	Leukocytosis, elevated CRP	Atrial fibrillation with AV block	No LVEF reported
Marx et al. [69]	Unstable	Normal	1st degree AV block	No LVEF reported
Kaczmarek et al. [70]	Stable	Leukocytosis	Ventricular tachycardia	No LVEF reported
Patel et al. [71]	Developed heart failure	NA	Complete block 6, AVB Mobitz type 8, Wenckebach 2, fluctuating AVB 12/16	LVEF—56.2%
Reznick et al. [72]	Stable	Anemia, high ESR and CRP	1st degree AV block > atrial flutter	No LVEF reported, left ventricular function, severe mitral regurgitation (3+) and a dilated left atrium
Cary et al. [73]	Unstable			No LVEF reported
Steere et al. [74]	Stable			No LVEF reported
	Stable	Leukocytosis	Complete 3rd heart block	LVEF—70%
	Stable	NA	Complete 3rd heart block	No LVEF reported
	Stable	Elevated ESR	Complete 3rd heart block, 1st degree AV block, Wenckebach block	LVEF—51%
	Stable		Complete 3rd heart block, 1st degree AV block, Wenckebach block	LVEF—72%
	Stable		Complete 3rd heart block, 1st degree AV block, Wenckebach block	No LVEF reported
	Stable		Complete 3rd AV block	No LVEF reported
	Stable		Complete 3rd AV block	No LVEF reported
	Stable		Complete 3rd heart block, 1st degree AV block, Wenckebach block	No LVEF reported
	Stable		Complete 3rd heart block, 1st degree AV block, Wenckebach block	No LVEF reported
	Stable		Complete 3rd heart block, 1st degree AV block, Wenckebach block	No LVEF reported No echo done
	Stable		1st degree AV block, Wenckebach	No LVEF reported
	Stable		1st degree AV block, Wenckebach	No LVEF reported
	Stable		1st degree AV block	LVEF-47%
	Stable		1st degree AV block	LVEF 62%
	Stable		1st degree AV block	LVEF 74%
	Stable		1st degree AV block	No LVEF reported
	Stable		1st degree AV block	LVEF 70%
	Stable		1st degree AV block	No LVEF reported
Muhammad et al. [75]	Stable	NA	1st degree AV block	No LVEF reported
Baron et al. [76]	Stable	NA	1st degree AV block	No LVEF reported
Büscher et al. [77]	Stable	NA	1st degree AV block	LVEF 72%
Rubin et al. [78]	Stable	NA	2nd degree AV block type 1 or type 2	No LVEF reported
Wagner et al. [79]	Stable	NA	Complete heart block	No LVEF reported
Kaczmarek et al. [80]	Stable	NA	2nd degree SA exit block showing typical Wenckebach periodicity with gradually shortening PP intervals	No LVEF reported
Celorio et al. [81]	Stable	NA	Complete AV block > Wenckebach > RBBB > LBBB	No LVEF reported
Kashou et al. [82]	Stable	NA	Complete 3rd AV block	No LVEF reported
Kashou et al. [82]	Stable	Elevated CKMB, ESR, proBNP, d-dimer	3rd degree AV block > 2nd degree type 2 > RBBB	No LVEF reported
Riaz et al. [83]	Stable	NA	Complete AV block	No LVEF reported
Legatowicz-Koprowska et al. [84]	Stable	NA	AV block type 2	No LVEF reported

AV—atrioventricular block, NA—not applicable, RBBB—right bundle branch block, LBBB—left bundle branch block, LVEF—left ventricular ejection fraction, ESR—erythrocyte sedimentation rate.

**Table 2:** Characteristics of atrioventricular blocks in men.

Variable	aOR [95% CI]	*p*-Value
Syncope	3.60 [1.31–9.96]	0.01
Time to resolution	1.37 [1.17–4.04]	<0.001
Variable arrhythmia	1.31 [1.08–2.16]	<0.001
Stability	1.82 [1.47–7.02]	0.04
Reversibility	1.51 [1.39–3.92]	0.01
Use of NSAIDs	1.07 [0.32–3.57]	0.75
Use of ceftriaxone	0.66 [0.25–1.80]	0.84
Use of doxycycline	0.33 [0.09–0.75]	<0.05

**Table 3:** Characteristics of atrioventricular blocks and other arrhythmias.

Variable	AV Block	Non-AV-Block	*p*-Value
	aOR [95% CI]	aOR [95% CI]	
Syncope	1.56 [1.51–4.76]	0.89 [0.79–1.01]	0.01
Time to resolution	0.01 [0.00–0.14]	0.82 [0.69–0.97]	<0.001
Variability	1.99 [1.47–8.31]	0.88 [0.75–1.04]	<0.001
Stability	1.27 [1.23–2.12]	0.96 [0.81–1.16]	0.01
Reversibility	1.57 [1.19–2.67]	0.95 [0.59–3.92]	0.01
Use of ceftriaxone	0.81 [0.24–2.69]	1.01 [0.91–1.20]	0.84
Use of doxycycline	2.88 [1.29–3.82]	1.17 [1.02–1.35]	<0.05

## Data Availability

Data is available on request addressed to the corresponding author.

## Supplementary Materials

The following supporting information can be downloaded at: https://www.mdpi.com/article/10.3390/jcdd11050131/s1, Table S1: Demographic and clinical characteristics, Table S2: Management and outcomes.
