# Supplementary material for: Associations of Atrioventricular Blocks and Other Arrhythmias in Patients with Lyme Carditis: A Systematic Review and Meta-Analysis
Source: J Cardiovasc Dev Dis. 2024 Apr 23;11(5):131. doi: 10.3390/jcdd11050131 (PMC11121903; doi:10.3390/jcdd11050131)
Supplement: Supplementary file 1 [file jcdd-11-00131-s001.zip › jcdd-2950331-supplementary.pdf]

Table S1. Demographic and clinical characteristics**.**

Authors	# of patients	Age	Gender	Comorbidities	Symptoms	Time to presentation	Total time to resolution of symptoms
Wan et al. [17]	5	35	5 males	NA	1-erythema migrans, 1- left facial nerve palsy, all symptoms	Acute	10 days
Zainal et al. [18]	1	46	Male	NA	Chest pain and dyspnea	3 week	2 weeks
Gazendam et al. [19]	1	22	Male	Asthma, drug abuse	Syncope	22 days	3 weeks
Esfandiari et al. [20]	1	26	Male	NA	Rash	1 week	3 weeks
Franco-Avecilla et al. [21]	1	56	Female	NA	Myalgia and syncope	15 days	2 weeks
Bamgboje et al. [22]	1	36	Male	NA	Fatigue, malaise, and myalgia	3 weeks	2 weeks
Zaid et al. [23]	1	19	Female	Chronic headaches, phthisis bulbi with secondary right eye blindness, pseudotumor cerebri, and severe obesity status post–laparoscopic sleeve gastrectomy	Headache and dyspnea	2 weeks	2 weeks
Arroja et al. [24]	1	42	Male	NA	Palpitations	2 days	2 weeks
Kannangara et al. [25]	1	37	Male	NA	Syncope	1 day	5 days
Rojas-Marte et al. [26]	1	19	Male	NA	Syncope	2 days	1 week
Khetpal et al. [27]	1	34	Female	NA	Syncope and shortness of breath	2 weeks	11 days
Wang et al. [28]	1	28	Male	NA	Syncope	4 days	2 days
Yoon et al. [29]	1	17	Male	NA	Viral syndrome	14 days	1 day
Dobbs et [30]	1	37	Female	NA	Numbness and tingling	5 days	14 days
Beach et al. [31]	1	17	Male	NA	Syncope	14 days	10 days
Shabbir et al. [32]	1	23	Male	NA	Palpitations	3 days	14 days
Kennel et al. [33]	1	20	Male	Cardiac rhabdomyosarcoma	Palpitations	2 days	1 day
Brunner et al. [34]	1	42	Male	NA	Palpitations	1 day	24 days
Greenberg et al. [35]	1	42	Male	NA	Fatigue and loose stool	2 days	14 days
Siebenlist et al. [36]	1	28	Male	NA	Syncope	3 weeks	10 days
Rostoff et al. [37]	1	49	Male	NA	Shortness of breath	5 days	15 days
Aringer et al. [38]	1	23	Male	NA	Oligoarthritis	1 day	14 days
Aringer et al. [38]	1	49	Male	NA	Oligoarthritis	7 weeks	4 weeks
Vasiljević et al. [39]	3	38,36,33	Female, Male, Male	NA	Syncope	3 weeks, 2 days, 7 days	2 weeks, 2 days, 1 month
Panic et al. [40]	1	36	Female	NA	Fatigue	10 days	12 days
Dam et al. [41]	1	44	Male	NA	Fatigue	5 days	15 days

Manek et al. [42]	1	20	Male	NA	Syncope	14 days	1 day
Nutt et al. [43]	1	20	Male	NA	Syncope	10 days	10 days
Khalil et al. [44]	1	24	Male	NA	Rash	3 weeks	2 days
Mayer et al. [45]	1	36	Female	NA	Rash	2 weeks	4 days
Jensen et al. [46]	1	45	Female	NA	Syncope	2 days	1 day
Jiménez-Castillo RA et al. [47]	1	23	Female	NA	Syncope	4 days	1 day
Lórincz et al. [48]	1	44	Female	NA	Rash	2 weeks	3 weeks
Vlay et al. [49]	1	67	Male	Smoking and hypertension	Rash	2 weeks	2 weeks
Munk et al. [50]	1	34	Male	NA	Syncope	11 days	14 days
Dernedde et al. [51]	1	47	Male	NA	Rash	11 days	5 days
Isath et al. [52]	1	22	Male	NA	Fever and rash	14 days	3 days
Timmer et al. [53]	1	45	Male	Smoking and alcohol use	Near syncope	4 days	3 days
Clinckaert et al. [54]	1	49	Male	Renal tumor s/p nephrectomy	Abdominal pain	A few weeks before	21 days
Zande et al. [55]	1	58	Male	NA	Rash	2 weeks	14 days
Matthiae et al. [56]	1	39	Male	NA	Palpitations	Several days	5 days
Xanthos et al. [57]	1	44	Male	Hyperlipidemia and smoking	Dizziness	21 days	3 days
Rosenfeld et al. [58]	1	31	Male	NA	Syncope, seizures and fever	2 days	7 days
Wenger et al. [59]	1	51	Male	NA	Chest pain and dyspnea	3 days	60 days
Bhattacharya et al. [60]	1	54	Female	NA	Syncope	3 weeks	2 days
Lo et al. [61]	1	63	Female	Hypertension	Dyspnea	7 days	21 days
Semmler et al. [62]	1	37	Male	NA	Dizziness	1 day	10 days
Chauhan et al. [63]	1	47	Male	NA	Syncope	3 days	21 days
Franck et al. [64]	1	66	Male	NA	Syncope	Unknown	19 days
Brownstein et al. [65]	1	31	Male	NA	Syncope	1 day	7 days
Prochnau et al. [66]	1	34	Male	NA	Syncope	14 days	5 days
Sempronі et al. [67]	1	37	Male	NA	Fever and rash	3 days	3 days
Konopka et al. [68]	1	41	Female	NA	Dyspnea	1 day	21 days
Marx et al. [69]	1	57	Male	NA	Fever and rash	7 days	None
Marx et al. [69]	1	49	Female	NA	Syncope and urinary incontinence	1 day	None
Kaczmarek et al. [70]	16	61.9	11 male and 5 female	HTN 12/16, T2DM 4/16, IHD 4/16, Afib 3/16, HF 2/16	Syncope	NA	None
Patel et al. [71]	1	59	Male	NA	Syncope	6 weeks	None
Reznick et al. [72]	1	39	Male	NA	Rash	2 weeks	None
Cary et al. [73]	1	31	Male	HTN, nephrolithiasis, appendectomy	Rash	3 months	None
Steere et al. [74]	19	30	Male	NA	Shortness of breath	NA	None

		36	Male	NA	Syncope	NA	None
		26	Male	NA	Palpitations	NA	None
		20	Female	NA	Syncope	NA	None
		19	Male	NA	Syncope, chest pain	NA	None
		22	Male	NA	Syncope	NA	None
		39	Male	NA	Palpitations	NA	None
		40	Male	NA	Chest pain	NA	None
		30	Male	NA	Dizziness	NA	None
		31	Female	NA	Palpitations	NA	None
		58	Male	NA	Palpitations	NA	None
		30	Male	NA	Palpitations	NA	None
		20	Female	NA	Palpitations	NA	None
		32	Male	NA	Palpitations	NA	None
		32	Female	NA	Palpitations	NA	None
		35	Male	NA	Palpitations	NA	None
		23	Male	NA	Palpitations	NA	None
		23	Male	NA	Palpitations	NA	None
		19	Male	NA	Palpitations	NA	None
Muhammad et al. [75]	1	55	Male	NA	Malaise	NA	None
Baron et al. [76]	1	30	Male	NA	Rash and dizziness	NA	None
Büscher et al. [77]	1	31	Female	NA	Syncope	NA	None
Rubin et al. [78]	1	52	Male	NA	Syncope	21 days	None
Wagner et al. [79]	1	30	Male	NA	Dizziness	5 weeks ago	None
Kaczmarek et al. [80]	1	48	Male	Dyslipidemia	Dizziness	Years ago	None
Celorio et al. [81]	1	36	Male	NA	Syncope	1 month	None
Kashou et al. [82]	1	26	Male	NA	Dizziness	1 month	None
Kashou et al. [82]	1	18	Male	NA	Dizziness	1 month	None
Riaz et al. [83]	1	27	Male	NA	Fever and rash	1 month	None
Legatowicz-Koprowska et al. [84]	1	60	Male	Atrial fibrillation	Dyspnea	1 week	None

HTN—hypertension, T2DM—Type 2 Diabetes Mellitus, IHD—Ischemic Heart Disease, Afib—Atrial Fibrillation, HF—heart failure, NA—not available

Table S2. Management and outcomes.

Authors	Treatment	Reversibility	Outcome
Wan et al. [17]	IV Ceftriaxone and oral doxycycline	Yes	All alive
Zainal et al. [18]	IV Ceftriaxone	Yes	Alive
Gazendam et al. [19]	IV Ceftriaxone	Yes	Alive
Esfandiari et al. [20]	IV Ceftriaxone, doxycycline and magnesium	No	Alive
Franco-Avecilla et al. [21]	IV Ceftriaxone	Yes	Alive
Bamgboje et al. [22]	IV Ceftriaxone and pacemaker	No	Alive
Zaid et al. [23]	IV Ceftriaxone	Yes	Alive
Arroja et al. [24]	IV Amoxicillin	Yes	Alive
Kannangara et al. [25]	IV Ceftriaxone and oral doxycycline	Yes	Alive
Rojas-Marte et al. [26]	IV Ceftriaxone	Yes	Alive
Khetpal et al. [27]	IV Ceftriaxone	Yes	Alive
Wang et al. [28]	Pacemaker	No	Alive
Yoon et al. [29]	Pericardiocentesis, CPR	No	Dead
Dobbs et [30]	IV Ceftriaxone	Yes	Alive
Beach et al. [31]	IV Ceftriaxone	Yes	Alive
Shabbir et al. [32]	IV Ceftriaxone	Yes	Alive
Kennel et al. [33]	None	Yes	Alive
Brunner et al. [34]	IV Ceftriaxone	Yes	Alive
Greenberg et al. [35]	IV Ceftriaxone	Yes	Alive
Siebenlist et al. [36]	IV Ceftriaxone	Yes	Alive
Rostoff et al. [37]	Pacemaker placement	Yes	Alive
Aringer et al. [38]	IV Ceftriaxone	Yes	Alive
Aringer et al. [38]	IV Ceftriaxone	Yes	Alive
Vasiljević et al. [39]	IV penicillin G	Yes	Alive
Panic et al. [40]	IV ceftriaxone and metronidazole	Yes	Alive
Dam et al. [41]	IV Ceftriaxone	Yes	Alive
Manek et al. [42]	IV Ceftriaxone and pacing	Yes	Alive
Nutt et al. [43]	IV Ceftriaxone and temporary pacemaker placement	Yes	Alive
Khalil et al. [44]	Pacemaker	Yes	Alive
Mayer et al. [45]	IV Ceftriaxone	Yes	Alive
Jensen et al. [46]	IV Ceftriaxone and pacemaker	No	Dead
Jiménez-Castillo RA et al. [47]	IV Ceftriaxone and pacing	No	Alive
Lórincz et al. [48]	Prednisone and atropine	No	Alive

Vlay et al. [49]	IV Ceftriaxone and pacemaker	No	Alive
Munk et al. [50]	IV Ceftriaxone and aspirin	No	Alive
Dernedde et al. [51]	IV Ceftriaxone	No	Alive
Isath et al. [52]	IV Ceftriaxone and pacemaker	Yes	Alive
Timmer et al. [53]	IV Ceftriaxone	No	Alive
Clinckaert et al. [54]	IV ceftriaxone and amiodarone	No	Alive
Zande et al. [55]	IV Ceftriaxone	Yes	Alive
Matthiae et al. [56]	IV Ceftriaxone	Yes	Alive
Xanthos et al. [57]	Doxycycline and pacemaker	Yes	Alive
Rosenfeld et al. [58]	Ceftriaxone, gentamicin, pacing	Yes	Alive
Wenger et al. [58]	Ceftriaxone and pacing	Yes	Alive
Bhattacharya et al. [60]	Ceftriaxone	Yes	Alive
Lo et al. [61]	Ceftriaxone	Yes	Alive
Semmler et al. [62]	Ceftriaxone	Yes	Alive
Chauhan et al. [63]	Ceftriaxone and pacing	Yes	Alive
Franck et al. [64]	Cefpodoxime	Yes	Alive
Brownstein et al. [65]	Ceftriaxone	Yes	Alive
Prochnau et al. [66]	Ceftriaxone	Yes	Alive
Semproni et al. [67]	Ceftriaxone, atropine, pacing	No	Dead
Konopka et al. [68]	Ceftriaxone and pacing	No	Alive
Marx et al. [69]	ACLS	No	Dead
Marx et al. [69]	ACLS	No	Dead
Kaczmarek et al. [70]	Pacemaker	No	Alive
Patel et al. [71]	Antibiotics	No>1st degree AV block remained	Alive
Reznick et al. [72]	Pacing and iV antibiotics	Yes	Alive
Cary et al. [73]	ACLS> had a concomitant tumor	No	Dead
Steere et al. [74]	Prednisone 9/18, Aspirin 9/18, Pacemaker needed in 6 and IV antibiotics in 8	Yes	Alive
		Yes	Alive
		Yes	Alive
		Yes	Alive
		Yes	Alive
		Yes	Alive
		Yes	Alive
		Yes	Alive
		Yes	Alive
		Yes	Alive
		Yes	Alive

		Yes	Alive
		Yes	Alive
		Yes	Alive
		Yes	Alive
		Yes	Alive
		Yes	Alive
		Yes	Alive
		Yes	Alive
Muhammad et al. [75]	Ceftriaxone	Yes	Alive
Baron et al. [76]	Ceftriaxone and temporary pacing>permanent pacemaker	No	Alive
Büscher et al. [77]	Doxycycline and ILR	Yes	Alive
Rubin et al. [78]	Cefuroxime and doxycycline	Yes	Alive
Wagner et al. [79]	Doxycycline	Yes	Alive
Kaczmarek et al. [80]	Ceftriaxone	No	Alive
Celorio et al. [81]	Ceftriaxone	No	Alive
Kashou et al. [82]	Ceftriaxone	Yes	Alive
Kashou et al. [82]	Doxycycline	Yes	Alive
Riaz et al. [83]	Ceftriaxone	Yes	Alive
Legatowicz-Koprowska et al. [84]	Ceftriaxone and doxycycline	Yes	Alive

IV—intravenous, ILR—implantable loop recorder
